# Pulmonary blastoma: a case report and brief review of the literature of tumor-induced hypoglycemia

**DOI:** 10.1186/s40200-016-0255-5

**Published:** 2016-08-15

**Authors:** Mohammad Reza Mohajeri Tehrani, Ali Ghorbani Abdehgah, Behnam Molavi, Salma Sefidbakht, Ali Reza Maleki, Amir Reza Radmard

**Affiliations:** 1 Endocrinology and Metabolic Research Center, Endocrinology and Metabolism Research Institute, Tehran University of Medical Sciences, Tehran, Iran; 2 Department of Surgery, Research Center for Improvement of Surgical Outcomes and Procedures, Shariati Hospital, Tehran University of Medical Sciences, North Kargar Street, Tehran, Iran; 3 Department of pathology, Shariati Hospital, Tehran University of Medical Sciences, Tehran, Iran; 4 Department of Radiology, Shariati Hospital, Tehran University of Medical Sciences, Tehran, Iran

**Keywords:** Tumor induced hypoglycemia, Pulmonary blastoma, Case report

## Abstract

**Background:**

Tumor induced hypoglycemia (TIH) is a rare clinical entity that can be caused by different mechanisms such as secretion of various substances, autoimmune disorders, massive tumoral infiltration of liver, and pituitary or adrenal glands destruction by tumors. Furthermore, any type of neoplasms can cause TIH.

**Case presentation:**

The patient presented with a case of classic biphasic pulmonary blastoma (CBPB) with hypoglycemia. Chest CT scan showed 2 huge masses in the right hemi-thorax and multiple smaller masses located in the left hemi-thorax. The patient underwent surgery, and was referred to an oncologist for adjuvant therapy.

**Conclusion:**

CBPB is a rare primary lung tumor with poor prognosis. They are classically large, symptomatic tumors with lymph node metastasis. Surgical resection at early stages has been more effective than other treatments; however, there is no standard treatment in unresectable cases. Adjuvant treatments have been temporarily effective.

## Background

Serum glucose levels of under 55 mg/dl, symptoms of hypoglycemia, and the disappearance of such symptoms after an increase in the serum’s glucose levels are indicative of hypoglycemia (Whipple’s triad) [1]. TIH is rare disorder mostly seen in elderly patients with advanced forms of cancer [2]. It is mainly observed in solid tumors with mesenchymal and epithelial origins [3].

Pulmonary Blastoma (PB) is mainly seen in young and middle aged patients with a history of smoking, it is rarely observed in elderly patients. Clinical symptoms of PB are hardly definitive or exclusive; they include coughing, chest pain, hemoptysis, fever, pleural effusion, asthenia, anorexia, weight loss and dyspnea [4–7]. In this report, a case of PB is reported that initially presented with symptoms of hypoglycemia.

Tumors of PB make up 0.25 to 0.5 % of primary lung tumors [5, 8]. They resemble infant primary lung tissues. The PB observed in adults is different from that observed in children from a histologic point of view; adult PB tissue includes portions of mesenchymal and epithelial tissue [4, 5].

There is no consensus on the histologic origins of PB; however, it is believed that such tumors originate in primitive multifunctional stromal lung blastoma. Watcher-Gropinger et al. illustrated that mutations in beta-catenin and p53 genes had significant roles in the appearance of these tumors [5].

In children, PB is similar to infantile rhabdomyosarcoma from an immunologic and cytogenetic perspective, suggesting that partial chromosome deletion may have a role in PB. However, there is no overall agreement regarding the cause of pulmonary blastoma and more investigation is needed [5].

In diagnosing PB, CX-Ray and CT scan can be used to observe lung masses. These masses may exhibit necrosis and parts of them can be enhanced using contrast [5].

## Case Presentation

**Fig. 1:**
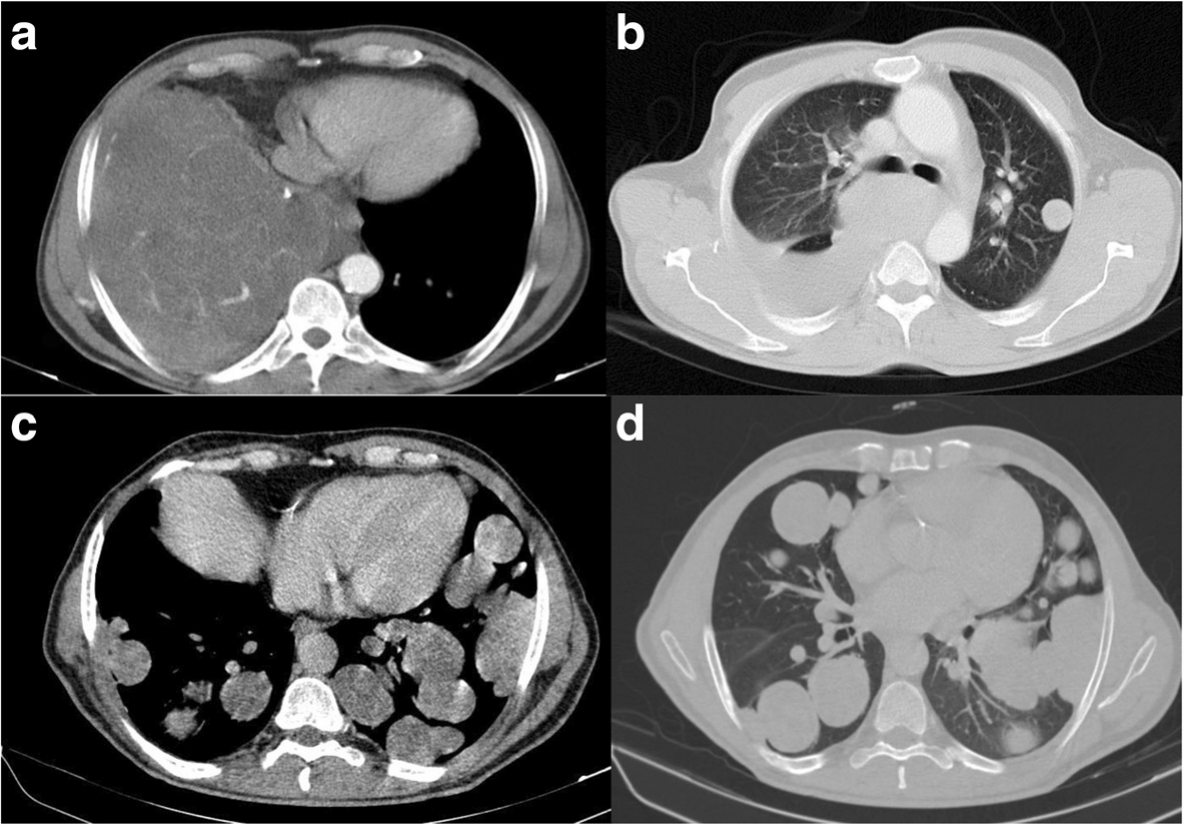
Contrast enhanced axial CT scan of the patient before **a**, **b** and after **c**, **d** surgery. There is a large solid mass in lower zone of right hemithorax with complete collapse of right lower lobe and cardiac displacement to left **a**. There is also an 18 × 15 mm parenchymal nodule in left upper lobe. Several other smaller nodules are found in this exam (not shown) in the rest of left lung. Two years after resection of the large mass in basal aspect of right hemithorax, there are numerous bilateral parenchymal and pleural-based nodules and masses with maximum size of 42 × 40 mm **c**, **d**

A 58-year-old male with history of weakness and malaise for 18 months was referred to Shariati hospital. In the initial work up, frequent episodes of hypoglycemia and elevated erythrocyte sedimentation rate were detected (Table 1). Chest CT scan showed 2 huge masses located in right hemi-thorax and multiple smaller masses located in the left hemi-thorax (Fig. 1a, b). The histopathological result of core needle biopsy before surgery was in favor of a solitary fibrous tumor. Right posterolateral thoracotomy and resection of 2 huge masses were performed and Permanent pathologic examination confirmed the diagnosis. After surgery, symptoms of hypoglycemia resolved.

**Table 1:** Laboratory parameters showed in first and last admission of patient which includes the primary evaluation in the first day of each admission

parameter	First admission	Second admission	Normal range
WBC	7260	8130	4000-10000 cumm
Hb	9.5	15.6	14–18 g/dl
Plt	405000	283000	150000–400000 cumm
ESR	116	77	0–20 mm/h
MCV	80	84	80–100 fl
BUN	16	12	7–21 mg/dl
Cr	1	0.9	0.7–1.4 mg/dl
BS random	62–59	42–30	mg/dl
Na	147	145	135–145 Meq/l
K	3.7	4	3.6–5.2 Meq/l
Ca	9.1	8	8.6–10.3 mg/dl
LDH	498	584	Up to 480 IU/l
C-peptide	0.05>	0.1	0.7–1.9 ng/ml
Cortisol (8 am)	14.5	-	5–23 micg/dl
IGF-1	33	-	22–197 ng/ml
Insulin	3.9	5.7	0.7–20 MIU/ml

**Fig. 2:**
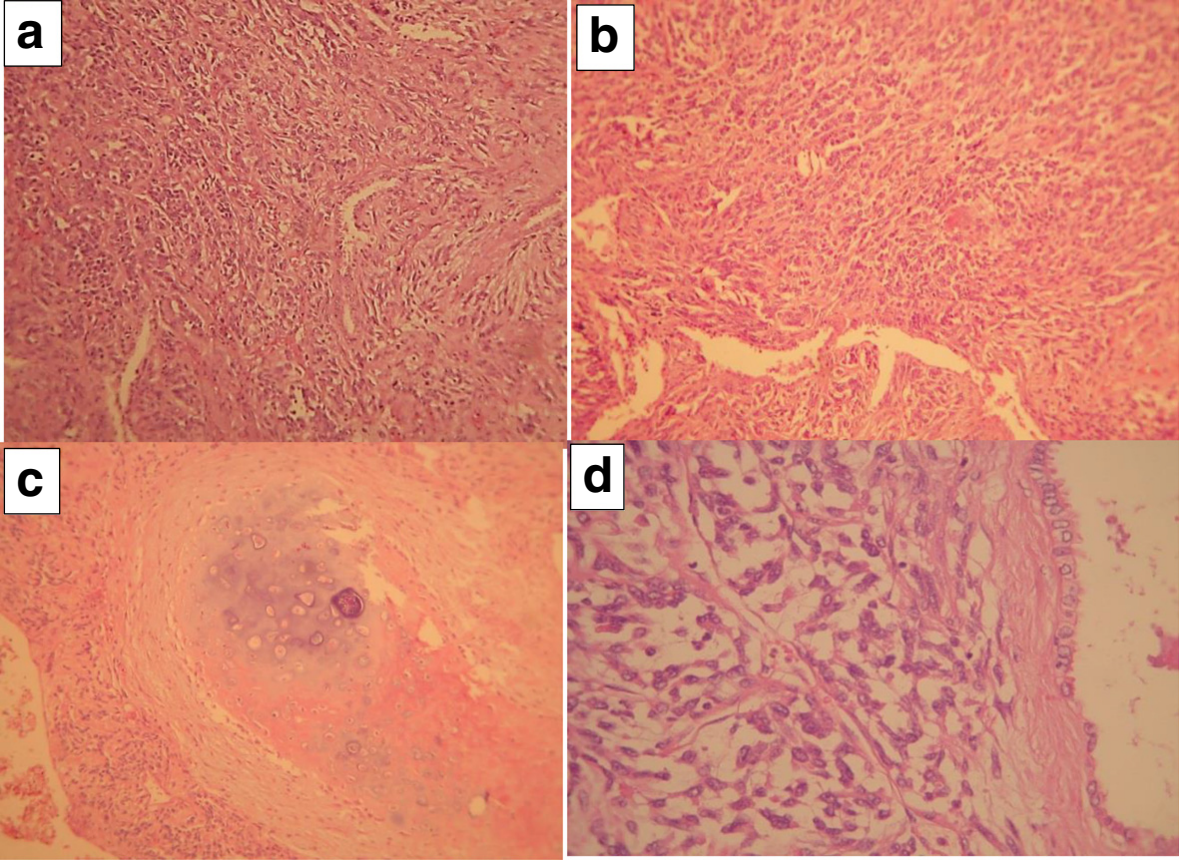
**a**: A biphasic tumor which composed of a mixture of malignant epithelial and stromal component (h&e. magnification × 40). **b**: The epithelial component was consisted of tubular and glandular structures, formed by columnar and cuboidal cells (h&e. magnification × 100). **c**: The stroma in some foci differentiated to cartilage (h&e. magnification × 40). **d**: The stroma of tumor was consisted of loose undifferentiated mesenchyme and areas with blastomatous appearance (h&e. magnification × 400)

**Fig. 3:**
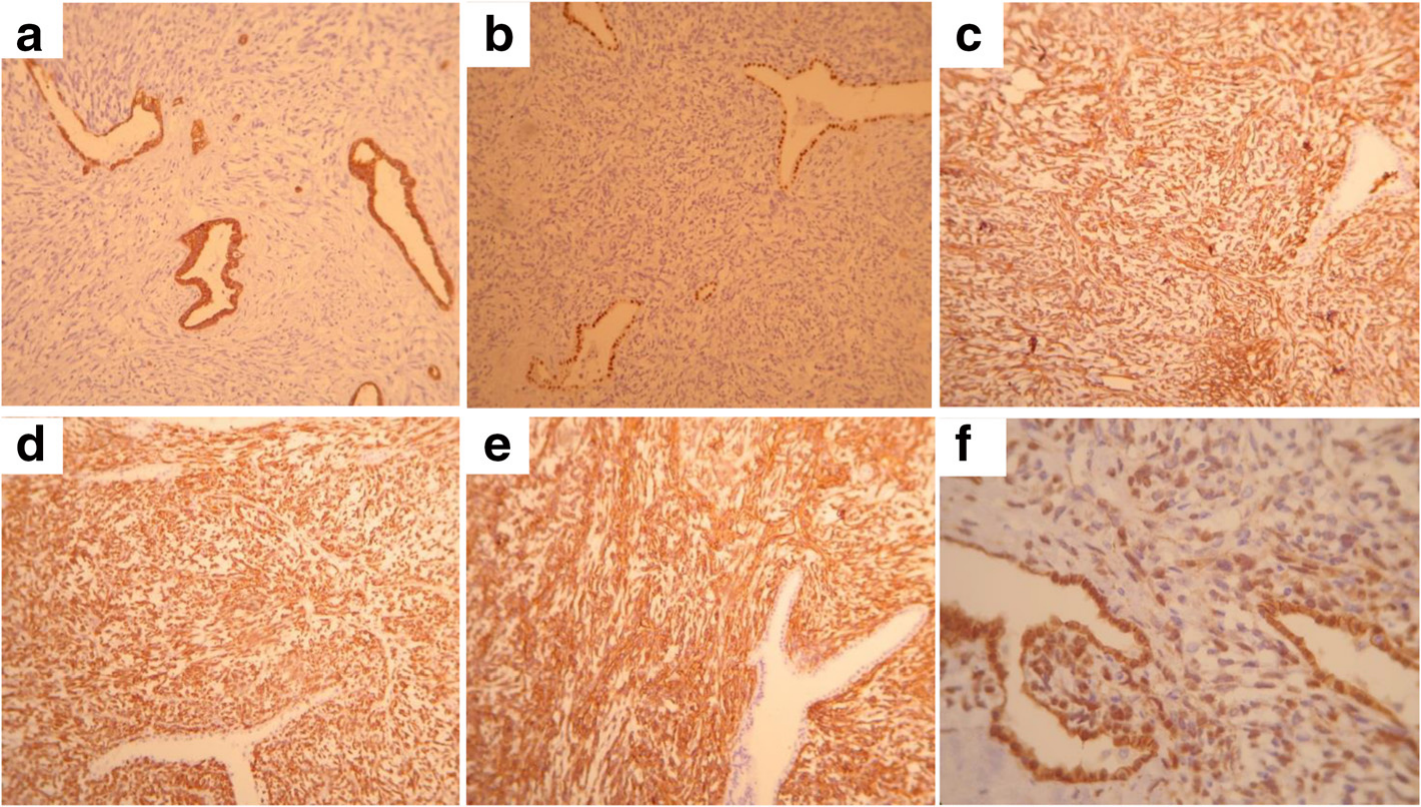
**a**-**b**: CK (AE1/AE3), and TTF-1 are positive in epithelial component. **c**-**e**: Vimentin, CD34 and bcl-2 are positive in mesenchymal component. **f**: β-catenin is expressed diffusely in both epithelial and mesenchymal elements

In spite of recommendations, the patient did not come back for resection of the left hemithorax masses. However, the patient visited our clinic due to hypoglycemic symptoms and dyspnea 2 years later. Imaging study revealed multiple bilateral parenchymal and pleural-based nodules (Fig. 1c, d). He underwent left posterolateral thoracotomy again and 45 masses were excised. Permanent pathology report revealed a pulmonary blastoma (Figs. 2, 3). His symptoms partially resolved and he was referred to an oncologist for adjuvant therapy after 32 days stay in ICU and surgical ward.

Afterwards the patient’s history and laboratory profiles were reviewed and histology of the right sided tumor was reassessed. It was determined that the correct diagnosis for the right hemi-thorax tumor was PB, similar to the diagnosis for the left hemi thorax tumor.

Tumors causing hypoglycemia can be classified according to their mechanism of action. The first group acts by secreting insulin, Insulinoma is the most prevalent example of this group. The second group acts through secreting insulin-like growth factor. The third group consists of multiple myeloma, lymphoma, and leukemia. The fourth group consists of metastatic neoplasms [1].

Tumors secreting insulin-like growth factor are also known as non-islet cell tumor hypoglycemia (NICTH); they can cause hypoglycemia through multiple mechanisms [1]. They mostly originate in lungs, the digestive system, adrenal gland, the pancreas, or the ovaries [9]. These groups of tumors frequently appear in the fifth or sixth decade of life. The average length of symptoms can range from a few weeks to a few months [7]. This patient was in sixth decade of life, when experienced an increase in insulin-like growth factor. Patient’s tumor was relatively large in all stages.

Hypoglycemia in these patients is mostly episodic. Each episode happens as a result of hunger, rather than eating food. Hypoglycemia in this patient was episodic and a result of hunger as well [7].

The possible pathophysiological causes of hypoglycemia include increases in secretion of IGF, invasion of the liver by a tumor, disruption of counter regulatory mechanisms as a result of adrenal blockage, increase on consumption of glucose by the tumor, and antibodies against insulin and insulin receptors [1, 9]. In this patient, higher secretions of IGF and an increase in glucose consumption due to the tumor were the causes of hypoglycemia.

Tumors that emit IGF2 (NICTHs) are diagnosed when the following are observed: low serum glucose levels during acute attacks, low levels of insulin and C-peptide in the serum, low levels of growth hormone and the insulin-like growth factor-1, normal or high levels of the insulin-like growth factor-2, and higher relative levels of insulin-like growth factor 2 compared to its counterpart similar to insulin-like growth factor 1 [1, 2, 10].

From a biochemical point of view, TIH is widely accompanied by hyperketonemia (<300 micromole/L). In 53 % of cases with NICTH hypokalemia is observed [7]. This patient had hypokalemia as well. Hypokalemia is caused by the insulin like activities of the insulin-like growth factor 2 (IGF2).

In all patients with epithelial or mesenchymal tumors that exhibit neuro psychiatric symptoms, NICTH should be considered [7]. Our patient reported weakness, tiredness, and excessive sweating. Furthermore, hypoglycemia was observed in the lab report.

NICTHs have a large domain. 40 % of them have mesenchymal origin; these include mesothelioma, hemangiopericytoma, and sarcomas. Another 40 % have epithelial origin; these include hepatocellular and lung sarcomas. The origins of the remaining 20 % of tumors are unknown [7].

PB is a distinct form of carcinosarcoma which was defined by Adluri [8]. It can appear as a parenchymal tumor or sub pleural nodules with pulmonary effusion [11]. In this report, the patient had multiple bilateral parenchymal and subpleural tumors.

PBs are divided into 3 groups according to their tissue make up. The first group is the monophasic group (good differentiation) which has an epithelial composition. The second group is the classic biphasic group, which consists of both epithelial and mesenchymal components (CBPB). The third group is the pleuropulmonary blastoma; they are tumors specific to childhood and only have mesenchymal tissue [12, 13]. This patient’s tumor was a CBPB.

Symptoms of CBPB are similar to symptoms of lower respiratory system infection [13]. In second visit, the patient’s chief complaint was dyspnea. However, episodic hypoglycemia was a secondary complaint as well. PB can invade the heart, the diaphragm, and the liver [7]. CX Ray and CT scan can assist in diagnosis. Functional imaging methods are inefficient at recognizing relapse and metastasis in NICTH [7]. Immunohistochemistry has a vital role in recognizing CBPB [9, 13]. Despite the new techniques for diagnosis, correct recognition before surgery is still difficult [4, 5, 7, 8, 12]. In this patient, accurate diagnosis was reached after the second surgery.

In both surgeries, the majority of the tumor was removed during the operation, resulting in the disappearance of hypoglycemia. Approximately 2 years after the first operation, the symptoms returned. However, the removal of the tumor during the second surgery resulted in the disappearance of symptoms again. The hypoglycemia was accompanied by low serum insulin, IGF-1, and C-peptide levels; Therefore, it may have been caused by increased glucose consumption of the large tumor and excretion of IGF-2 by the tumor. However, it is not possible to confirm this hypothesis at this time, as there were no methods available to measure blood IGF-2 levels.

Medical treatments for NICTH include diazoxide, octreotide, glucagon infusion, steroids, and recombinant growth hormone. Glucose tablets can be prescribed as well [9]. However, the definitive treatment is surgery [4, 5, 12].

Surgical treatments for NICTH include complete removal of the tumor, or debulking accompanied with radiotherapy and chemotherapy [5, 7, 10].

Glucocorticoids are the most effective method of long term management [1, 7]. The hypoglycemia can be controlled through chemotherapy, radiotherapy, and embolization with various success rates [9]. PBs are fast growing tumors with poor prognosis [4, 6]. Factors that illustrate the poor prognosis in adult PBs include relapse, metastatic illness, larger than 5 cm tumor, and lymphatic system involvement [6, 7, 13].

The effectiveness of chemotherapy and radiotherapy is unknown. However, due to the metastatic nature of this patient’s illness, he was referred for adjuvant treatment. In previous reports chemotherapy has been recommended in cases of metastasis [4, 7]. Overall, studies regarding the effects of chemotherapy and radiotherapy are scarce, but some researchers have reported the effectiveness of using platinum [13].

In locally advanced tumors, mediastinal lymph node involvement, or metastatic cases chemotherapy / radiotherapy can be used. Chemotherapy’s effect on treatment is limited [3, 4].

In our patient, 40 months have passed since the beginning of illness. After 2 surgeries the tumor is currently under control with the help of chemotherapy.

## Conclusion

In this patient, symptoms of hypoglycemia disappeared after 2 tumor debulking surgeries. Furthermore, serum insulin, C-peptide, and IGF1 were normal after the operation. Despite the fact that it was not possible to measure IGF2 levels at our center, it is possible to assume with some measure of certainty that the cause of hypoglycemia in this case was the patient’s tumor. However, absolute certainty would require further biochemical and molecular studies.

It has become increasingly apparent in the recent years, that almost any type of neoplasm can cause hypoglycemia by a number of mechanisms.

NICTH is a rare cause of hypoglycemia usually seen in large mesenchymal tumors. CBPB is characterized by both epithelial and mesenchymal malignant components. Diagnosis of CBPB with core needle biopsy is difficult. If histopathologic examinations revealed the diagnosis of solitary fibrous tumor in a patient with multiple lung tumors not compatible with patient’s clinical and imaging findings, repeated histopathological evaluation is recommended. The principal treatment for CBPB is surgery. Chemotherapy and radiotherapy have been tried for advanced tumors.

## Abbreviations

CBPB, Classic Biphasic Pulmonary Blastoma; IGF, Insulin-like Growth Factor; NICTH, Non-Islet Cell Tumor Hypoglycemia; PB, Pulmonary Blastoma; TIH, Tumor Induced Hypoglycemia

## References

[1] Iglesias P, Díez JJ (2014). Management of endocrine disease: a clinical update on tumor-induced hypoglycemia. Eur J Endocrinol.

[2] Pelosof LC, Gerber DE (2010). Paraneoplastic syndromes: an approach to diagnosis and treatment. Mayo Clinic Proceedings.

[3] Sakata S, Saeki S, Hirooka S, Hirosako S, Ichiyasu H, Kohrogi H (2015). A Case of Biphasic Pulmonary Blastoma Treated with Carboplatin and Paclitaxel plus Bevacizumab. Case Rep Oncological Med.

[4] Sharma A, O’GORMAN K, Aman C, Rassl D, Mohamid W, Polychronis A (2013). A rare occurrence of biphasic pulmonary blastoma in an elderly male. Anticancer Res.

[5] Wang Y-X, Zhang J, Chu X-Y, Liu Y, Li F, Wang Z-B (2014). Diagnosis and multi-modality treatment of adult pulmonary plastoma: Analysis of 18 cases and review of literature. Asian Pac J Trop Med.

[6] Kilic D, Yilmaz C, Tepeoglu M, Vural C, Caner H (2015). Biphasic Pulmonary Blastoma Associated with Cerebral Metastasis. Turk Neurosurg.

[7] Dutta P, Aggarwal A, Gogate Y, Nahar U, Shah VN, Singla M (2013). Non-islet cell tumor-induced hypoglycemia: a report of five cases and brief review of the literature. Endocrinology Diabetes Metabolism Case Reports.

[8] Adluri RKP, Boddu SR, Martin-Ucar A, Duffy JP, Beggs FD, Morgan WE (2006). Pulmonary blastoma—a rare tumor with variable presentation. Eur J Cardiothorac Surg.

[9] Thomas J, Kumar SC (2013). Nonislet cell tumor hypoglycemia. Case Rep Endocrinol.

[10] Bodnar TW, Acevedo MJ, Pietropaolo M (2013). Management of non-islet-cell tumor hypoglycemia: a clinical review. J Clin Endocrinol Metab.

[11] Lee TH, Lee KY, Kim SR, Min KH, Park SJ, Lee HB (2007). A Case of Huge Pulmonary Blastoma With Multiorgan Invasion. Tuberc Respir Dis.

[12] Xiu Y, Jiang L, Liu W (2015). Classic biphasic pulmonary blastoma with brain and axillary metastases: a case report with molecular analysis and review of literature. Int J Clin Exp Pathol.

[13] Alahwal MS, Maniyar IH, Saleem F, Alshiekh M (2012). Pulmonary blastoma: a rare primary lung malignancy. Case Rep Med.

